# Spatiotemporal Root-Trait Plasticity Underpins Almond Yield Stability and Enhanced Water and Nitrogen Use Efficiency Under Prolonged Fertigation Reduction

**DOI:** 10.3390/plants15030409

**Published:** 2026-01-29

**Authors:** Shuangxi Zhou, Alexandra Lawlor, Rob R. Walker, Everard J. Edwards

**Affiliations:** CSIRO, Waite Campus, Urrbrae, SA 5064, Australiarob.walker@csiro.au (R.R.W.); everard.edwards@csiro.au (E.J.E.)

**Keywords:** climate resilience, farm management, resource use efficiency, root phenotyping, root system resilience, soil management

## Abstract

The root system provides the interface between the plant and the soil that is responsible for water and nutrient uptake and transport. We hypothesized that almond trees in the commercial production environment could adjust their root acquisitive traits with distance vertically and horizontally from driplines as adaptive responses to within-orchard reductions in irrigation and nitrogen inputs. We compared the responses of root acquisitive traits under four years of treatments ranging from +W+N (15 ML ha^−1^ water and 300 kg ha^−1^ nitrogen per season) to −W−N (10.5 ML ha^−1^ water and 160 kg ha^−1^ nitrogen per season, with −W involving a 30% reduction in irrigation and −N involving a 46% reduction in nitrogen). Roots (<3 mm) were sampled through soil coring in the winters of 2017, 2018, and 2019. Root sampling was conducted along the vertical gradient and along the horizonal gradient (0 cm, 80 cm, and 240 cm from the dripline). Four years of treatments highlighted that the data variation was driven mainly by the difference between the +W and −W treatments (along PC1). Further, the difference between −W−N (combined resource reduction) and the other three treatments (+W+N, +W−N, and −W+N) contributed to the data variation (along PC2). Also, the temporal dynamics of treatment effects over 2017, 2018, and 2019 suggested a temporally strengthened +W−N effect to increase root biomass, average root diameter, specific root surface area (SRA), and specific root length (SRL) at deeper soil depths and at greater soil distances from driplines. These findings on the spatial and temporal plasticity of traits representing root resource acquisition capabilities highlighted the important role of root systems in maintaining crop productivity under reduced irrigation and nitrogen inputs.

## 1. Introduction

The distribution and morphology of roots in the root zone are key factors that shape tree capacity for uptake of water and nutrients and their effect on tree performance and lifespan. When challenged by adverse soil characteristics, such as soil compaction, water deficit, or high soil conductivity (whether through nutrient application or salinity), the root system must continue to meet as much as possible of the plant’s resource requirements [1]. Consequently, the capacity of a tree to take up these resources is primarily controlled by factors such as the extent of roots, the speed with which new roots are produced and grow, and how long they live. Effective irrigation to keep the root zone wet for fruit trees (e.g., almond and avocado), particularly in advance of the critical growth stages or before dry and hot weather events, has long been recognized as important by Australian and international growers.

To better understand tree crop resilience and how it may be affected by changes in orchard management practices, e.g., changes in irrigation or fertilizer application strategies, it is imperative to have the best possible understanding of the key characteristics of the root system. Root system resilience under changes to management practice has critical implications for nutrient uptake, tree stability, and ultimately yield. For perennial tree crop production systems, the pattern, timing, and total amount of root production will potentially vary substantially from season to season. Field observations of root function and phenology have the potential to greatly improve our understanding of how irrigation and fertilizer application can best be utilized by trees and how this affects orchard performance. A better understanding of the location of active fine roots in the soil, the timing of root growth flushes, and the impact of root system phenology through the growing season has the potential to improve the accuracy, in both time and space, of fertilizer and irrigation inputs in almond orchards.

Compared to other tree crops, almond trees require a relatively higher water supply for optimal growth and fruit production, making efficient water use essential for the almond industries worldwide. Efficient fertigation practices will enable orchard productivity and profitability to remain resilient to climatic extremes, such as increased severity and frequency of droughts and consequent reduced water availability [2]. Improvements to irrigation practices can provide growers with the capacity to efficiently and effectively manage production costs, maintain consistent yield and quality, and secure profitability, particularly in the context of climate change. In the case of almonds, achieving an absolute increase in production is not fully effective if not matched by an improvement in production efficiency. Improving production efficiency and reducing costs, for example, by maximizing irrigation and fertilizer use efficiency, are key challenges for the industry. A better understanding of the scope and functionality of the root system is required as a basis for improving production efficiency and for reducing the variability in kernel yield, for example, of Nonpareil trees [3]. Specific root surface area (SRA) and specific root length (SRL) are usually used to compare root resource acquisition (i.e., benefit) against root construction and maintenance (i.e., cost). Both parameters represent a species’ underground resource strategy [4,5]. Further, root diameter is a proxy of root hydraulics and root lifespan [6]. In addition, SRA, SRL, and average root diameter can reflect the fineness of the root system under environmental changes [5] and usually show a high degree of plasticity in response to environmental changes [4]. Fine-root acquisitive traits (i.e., SRA and SRL) and their dynamics are important for understanding the resource acquisition strategy of fruit and nut trees, particularly in the context of drier and warmer climate scenarios. In the commercial orchard environment, fertigation is usually applied through driplines, and the water and nutrients are mainly located at the topsoil, where crop types such as almond and avocado allocate most of their roots. The investment by trees into allocating resources for root growth in soil horizontally and vertically from driplines is driven by a need to explore and access water and nutrients from such zones, but at the same time it could impose resource limitations on fruit trees.

Despite the significance of the afore-mentioned root traits, the study of root systems in commercial almond orchards is difficult and time-consuming, which leads to their very poor representation in research. For other crops, previous studies have reported the seasonal dynamics of root length distribution in response to temporal fluctuations in soil moisture [7,8,9]. Orchard-based studies on root acquisitive traits, their horizontal (besides vertical) responsiveness, and their inter-seasonal heterogeneity are limited. Identifying the cause of any such inter-seasonal variability, together with any relationship with water and nitrogen availability, will improve our understanding of the resilience of root systems and assist in developing optimized irrigation and fertilizer application strategies.

For *Prunus* hybrids, glasshouse studies have found that these root acquisitive traits are sensitive to environmental stresses and that the trait responses are hybrid-dependent and multi-dimensional [10,11]. Further, the stress responses of root growth and root acquisitive traits (e.g., SRA) have been reported to be correlated with each other [11]. Besides the spatial variability of root distribution, better understanding of the inter-seasonal variability of root distribution is also important, as the performance of almond trees in a given year can be limited by the irrigation reduction during and after the harvest of the previous year [3,12]. However, evidence from commercial almond orchards is very limited, and inter-seasonal field studies are rare, largely due to the logistic challenge of studying roots in the field.

**Figure 1:**
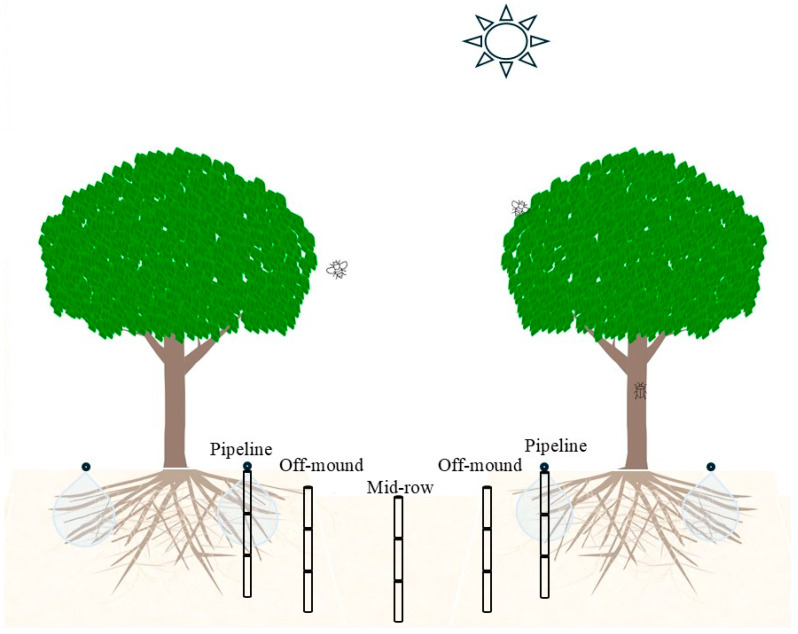
Description of the four-year field experiment on mature Nonpareil trees grafted on the Nemaguard rootstock at the commercial farm at Lindsay Point, Victoria, Australia: layout of soil coring positions (right) along the vertical gradient (D1: 0–25 cm under the dripline; D2: 25–50 cm under the dripline; D3: 50–75 cm under the dripline) and the horizonal gradient (dripline: 0 cm from the dripline; off-mound: 80 cm from the dripline; mid-row: 240 cm from the dripline).

In this study, we hypothesized that, under water and/or nitrogen reductions, almond trees in commercial environments could modify their root acquisitive traits in a similar manner to that reported on saplings in glasshouse environment [10]. Our objective was to test this hypothesis and the spatial and temporal dynamics of the root adjustments, if any, with a four-year field experiment. The experiment included four treatments (i.e., commercial standard practice, a 30% reduction in irrigation, a 46% reduction in nitrogen application, and a combination of the two; Figure 1), where the treatment-induced adjustments of root traits and tree water use in a commercial production environment were compared.

## 2. Results

### 2.1. Tree Water Use

For the four seasons, the percentage of reduction in tree water use was less than the percentage of reduction in applied irrigation. In general, very high sap flow rates were recorded across the four treatments, with sap velocity typically exceeding 25 cm hr^−1^ and whole-tree sap flow rates in excess of 2.5 L hr^−1^ in the middle of the day once the canopy was established. There was a minor reduction in tree water use in the −W treatments, but there were no significant differences among treatments. The responsiveness of the canopy to changes in the evaporative demand of the atmosphere, represented by reference evapotranspiration (ETo), was apparent, as was the gradual increase in tree water use through September and October as the canopy grows. Total seasonal tree water use ranged from 9 to 17 kL per tree for the post-budburst to harvest period, equating to 3.1 to 5.9 ML ha^−1^.

### 2.2. Root Biomass

For the four seasons, minimal impacts of the reduced water and/or nitrogen inputs on fine root production were observed. The root biomass was concentrated mainly in the top 25 cm of soil. The soil coring during winter disclosed limited treatment effects on root biomass, with a small increase in biomass in the reduced input treatments (i.e., −W and −N). There was no statistically significant difference in root biomass between the treatments in any of the years, with the largest increase being 8%, caused by the +W−N treatment, across the four years of treatments.

### 2.3. PCA Analysis of Root Traits

**Figure 2:**
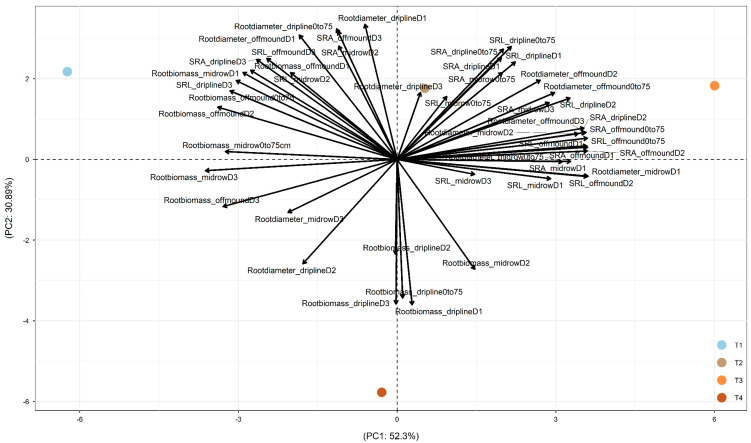
PCA depicting the differential responses of root traits under four treatments (T1: +W+N, T2: +W−N, T3: −W+N, T4: −W−N) along the vertical gradient (D1: 0–25 cm under the dripline; D2: 25–50 cm under the dripline; D3: 50–75 cm under the dripline; ‘0–75 cm’: the total of D1, D2, and D3) and along the horizonal gradient (dripline: 0 cm from the dripline; off-mound: 80 cm from the dripline; mid-row: 240 cm from the dripline) at the commercial farm at Lindsay Point, Victoria, Australia. Root traits included the specific root surface area (SRA), specific root length (SRL), average root diameter, and root biomass. Data were collected on roots (≤3 mm) from soil coring in the winter of 2017 (after 2 years of treatments). PC1 explained 52.3% of the total variation, and PC2 explained 30.89% of the total variation. PC1 and PC2 explained 83.19% of the total variation.

In 2017, after two years of treatments, PC1 was driven by the difference between +W+N (on the left part) and the other three treatments (Figure 2). PC2 was driven by the difference between −W−N (on the bottom part) and the other three treatments (on the top part). +W+N led to root exploration in deeper soil and in soil more distant from the dripline through (1) increased root biomass at off-mound D1, D2, and D3 and mid-row D1, D2, and D3; (2) increased root diameter at dripline D3, off-mound D2 and D3, and mid-row D2; and (3) increased SRA and SRL at driplines D1 and D2, off-mound D1 and D2, and mid-row D1 and D3. Also, −W−N increased root biomass at driplines D1, D2, and D3 (Figure 2).

**Figure 3:**
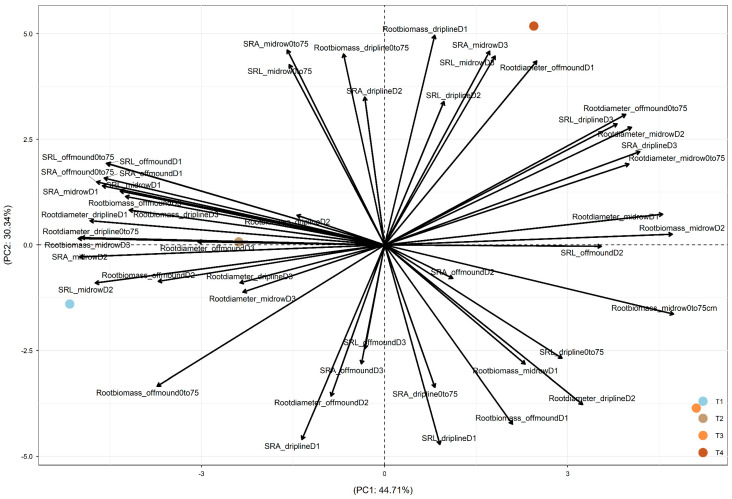
PCA depicting the differential responses of root traits under four treatments (T1: +W+N, T2: +W−N, T3: −W+N, T4: −W−N) along the vertical gradient (D1: 0–25 cm under the dripline; D2: 25–50 cm under the dripline; D3: 50–75 cm under the dripline; ‘0–75 cm’: the total of D1, D2, and D3) and along the horizonal gradient (dripline: 0 cm from the dripline; off-mound: 80 cm from the dripline; mid-row: 240 cm from the dripline) at the commercial farm at Lindsay Point, Victoria, Australia. Root traits included the specific root surface area (SRA), specific root length (SRL), average root diameter, and root biomass. Data were collected on roots (≤3 mm) from soil coring in the winter of 2018 (after 3 years of treatments). PC1 explained 44.71% of the total variation, and PC2 explained 30.34% of the total variation. PC1 and PC2 explained 75.05% of the total variation.

In 2018, after three years of treatments, PC1 was driven by the difference between +W (+W+N and +W−N, the left part of PC1, showing similar effects) and −W (−W+N and −W−N, on the right; Figure 3). PC2 was driven by the difference between −W+N (in the bottom part) and −W−N (in the top part). Both +W+N and +W−N led to root adjustments in deeper soil and more distant soil through (1) increased root biomass at driplines D2 and D3, off-mound D2 and D3, and mid-row D3; (2) increased root diameter at driplines D1 and D3, off-mound D3, and mid-row D3; and (3) increased SRA and SRL at off-mound D1 and D3 (which drove their increase at 0–75 cm) and mid-row D1 and D2 (which drove their increase at 0–75 cm). Additionally, −W−N increased SRA and SRL at dripline D2 and mid-row D3 (Figure 3).

**Figure 4:**
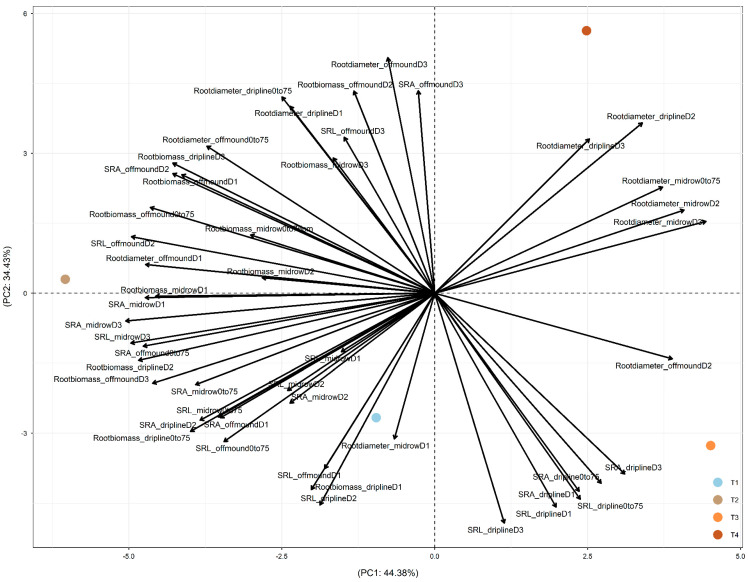
PCA depicting the differential responses of root traits under four treatments (T1: +W+N, T2: +W−N, T3: −W+N, T4: −W−N) along the vertical gradient (D1: 0–25 cm under the dripline; D2: 25–50 cm under the dripline; D3: 50–75 cm under the dripline; ‘0–75 cm’: the total of D1, D2, and D3) and along the horizonal gradient (dripline: 0 cm from the dripline; off-mound: 80 cm from the dripline; mid-row: 240 cm from the dripline) at the commercial farm at Lindsay Point, Victoria, Australia. Root traits included the specific root surface area (SRA), specific root length (SRL), average root diameter, and root biomass. Data were collected on roots (≤3 mm) from soil coring in the winter of 2019 (after 4 years of treatments). PC1 explained 44.38% of the total variation, and PC2 explained 34.43% of the total variation. PC1 and PC2 explained 78.81% of the total variation.

In 2019, after four years of treatments, PC1 was driven by the difference between +W (+W+N and +W−N, on the left of PC1, mainly driven by the effect of +W−N) and −W (−W+N and −W−N, on the right; Figure 4). PC2 was driven by the difference between −W−N (in the top part) and the other three treatments (in the bottom part). +W−N led to root adjustments in deeper soil and more distant soil through (1) increased root biomass at driplines D2 and D3; off-mound D1, D2, and D3; and mid-row D1, D2, and D3; (2) increased root diameter at dripline D1 and off-mound D1 and D3; and (3) increased SRA and SRL at off-mound D1 and D2 and mid-row D1, D2, and D3 (Figure 4).

Overall, across 2017, 2018, and 2019, PC1 was mainly driven by the difference between +W and −W, and PC2 was mainly driven by the difference between −W−N (i.e., combined irrigation and nitrogen reductions) and the other three treatments (+W+N, +W−N, and −W+N). Also, the temporal dynamics of root-trait adjustments over 2017, 2018, and 2019 showed the temporally increased impact of +W−N treatment on root biomass, diameter, SRL, and SRA (i.e., along PC1).

## 3. Discussion

This study collected a unique data set over four continuous seasons on the spatial (i.e., vertical and horizontal) and temporal responsiveness of root acquisitive traits under a 30% reduction in irrigation and/or a 46% reduction in nitrogen in a commercial orchard. The results highlighted the critical role that root system resilience can play in the stability of orchard productivity under reduced water and nitrogen inputs. For the four seasons, minimal impacts of the reduced water and/or nitrogen inputs on fine root production were observed. These results were consistent with those on almond yield, which showed no significant treatment effects [13]. The vertical, horizontal, and temporal responsiveness of root acquisitive traits suggested whole-root-system coordination in carbon allocation and trait plasticity. These findings highlight the resilience of the almond root system through its capacity to maintain adequate supply of water and nitrogen to the shoots even under a 30% reduction in irrigation and/or a 46% reduction in nitrogen application.

### 3.1. Tree Water Use Driven by Canopy Evaporative Demand

Soil water stress can develop quickly in almond trees if root-zone water content is limited while there is a high evaporative demand [3]. Increased root biomass and extended root zones (i.e., vertically and horizontally; Figure 2, Figure 3 and Figure 4; Supplementary Figures S1–S3) can facilitate tree exploration for resources in soil space, which is particularly important when the canopy evaporative demand is high.

The high sap flow rates resulted in some difficulties in maintaining reliable data during the middle of the day, particularly in 2018/19, leading to some underestimates in the data and therefore more variation in the results than was expected. Furthermore, the data presented were calculated with average scaling values and did not account for possible small differences in tree architecture properties (i.e., canopy size) between trees. Nonetheless, the data collected were generally well correlated with atmospheric conditions and canopy growth. There is a gradual increase in tree water use through September and October as the canopy grows. Light interception data for the same trees under the same treatments, reported by Treeby [13], generally indicated that peak light interception occurred in December.

Daily tree water use exhibits a strongly curvilinear relationship with ETo-. This was seen with all the sensors across all three seasons and was not due to a limitation of the sensors to measure maximum flow during the summer. The irrigation applied to the site averaged 14.6 ML ha^−1^ for the entire growing season, whereas the values for tree water use quoted from the sap flow meter (equating to 3.1 to 5.9 ML ha^−1^) were for part of the season (post-budburst to harvest), and, further, water losses due to soil evaporation or deep drainage were not measured. Moreover, sap flow sensors require accurate scaling for each installation and commonly provide an underestimate in comparison to weighing lysimeters. Averaged over the 2016/17 and 2017/18 seasons, the reduced irrigation treatments resulted in a 20% decrease in growing-season tree water use.

Zhou et al. [10] reported the covariation of hydraulics between the canopies and root systems of *Prunus* genotypes under reduced water availability. The sap flow data show that the percentage reduction in tree water use under the −W treatments was less than the percentage reduction in irrigation, suggesting an improvement in water use efficiency by those trees. These findings also support the conclusion of Treeby [13] that the reduction in irrigation applied does not drive a statistically significant reduction in yield over the four seasons studied.

### 3.2. Vertical and Horizontal Responsiveness of Root Biomass and Root Acquisitive Traits

Root-trait adjustments under orchard management changes have important implications for understanding the roles of root system resilience underlying fruit tree performance. In this study, improved water and nitrogen use efficiency tended to be related to the spatial trait adjustments of the roots. This connection indicates that root plasticity could play an important role in the underlying mechanism which allows plants to maintain water demand and, ultimately, yield under reduced resource inputs.

Plant biomass allocation towards roots in deeper soil layers has been reported to be enhanced with higher nitrogen application [14]. Meanwhile, traits of roots in deeper soil layers have been reported to be highly correlated with plant water consumption [15]. However, studies on the spatial and temporal dynamics of root responses under combined water and nitrogen manipulation conditions are limited and much needed [16,17]. This study provided inter-seasonal evidence in addressing this question and highlighted the importance of quantifying the responses of other stress-sensitive organs beyond leaves, such as roots, for the comprehensive understanding of stress adaptation at the whole-plant scale [18].

This study, to the best of our knowledge, is the first in a commercial almond orchard to evaluate the spatial (vertical and horizontal) and temporal response patterns of root acquisitive traits (SRA, SRL, and root diameter) under irrigation and fertilizer reduction treatments. Four years of treatments highlighted that the data variation was mainly driven by the difference between the +W and −W treatments (along PC1 in Figure 2, Figure 3 and Figure 4). To a lesser extent, the difference between −W−N (combined resource reduction) and the other three treatments also notably contributed to the data variation (along PC2 in Figure 2, Figure 3 and Figure 4). The temporal dynamics of root-trait adjustments over 2017, 2018, and 2019 suggested an increased impact of the +W−N treatment on root biomass, diameter, SRL, and SRA (along PC1). These results suggested that almond trees in the commercial production environment can adjust root traits to explore resources in the soil space (i.e., water or nitrogen), both vertically (0–25 cm, 25–50 cm, and 50–75 cm) and/or horizontally from the dripline to the off-mound zone (80 cm from the dripline) and mid-row zone (240 cm from the dripline).

In this study, water reduction appears to have driven the adjustment of root traits (i.e., thicker roots and deeper-soil vertical allocation of roots) towards enhancing the resource-acquisition behavior of almond root system. When comparing the treatment effect, the effects of water reduction treatments (along PC1) appeared to be larger than the nitrogen reduction treatments in explaining the majority of variation among root-trait adjustments. This may be because water reduction generally exerts stronger pressure due to its immediate physiological impacts relative to nitrogen reduction. Higher nitrogen application has been reported to enhance root morphological development under moist conditions but restrict root growth under drought conditions [19,20].

The study also contributed to enhancing our understanding of how plants balance root diameter, SRL, SRA, and biomass allocation vertically (i.e., along soil depth) and horizontally (i.e., lateral spread) to explore soil resources. This study identified positive correlations among root biomass, average root diameter, SRA, and SRL, indicating that, at a whole-root-system level, +W promoted carbon investment into root biomass at deeper soil depths and at greater soil distance from driplines. This study supports Ceolin et al. [1], who reported that the plant root system can economize root carbon investment vertically to explore soil resources.

Furthermore, this study offers evidence proving that the almond root system can also economize root carbon investment horizontally. Such coordinated responses at the entire root system level could be part of a whole-tree strategy to efficiently partition and target carbon allocation wherever soil resources are accessible [1]. To further test this hypothesis at the whole-tree level, the canopy-level responses and their trade-offs with the root system responses will need to be investigated in the commercial production environment [10,11]. Predicting the long-term consequence of environmental changes on fruit crop plantings requires improved understanding of the coordination between canopy and root systems. *Prunus* genotypes have been reported to be highly capable of modifying their leaf and root acquisitive traits in response to reduced water availability [10]. For *Prunus* plants, leaf mass per unit leaf area could be increased when photosynthetically active radiation was increased and when a 30% irrigation reduction was applied [10]. When the water or nitrogen applications were reduced, light penetration of the canopy was improved, particularly in the lower parts of the canopy [13], for trees under the same −W and −N treatments as those used in this study. The results suggest that the almond tree root systems were especially resilient to the reduced W and N inputs and were able to maintain the canopy demand for W and N. Indeed, this is supported by the lack of overall yield impacts observed at the same experimental plots over the four seasons [13].

The vertical, horizontal, and temporal dynamics of root responsiveness highlighted in this study are insightful for future studies on crop resilience under environmental changes, particularly in Australian orchard production systems. Future studies disentangling the complex range of sensing and signaling mechanisms underlying water and nitrogen resource acquisition, transport, and allocation strategies could improve our current understanding at the eco-physiological scale. It would also be interesting for future studies to compare the root plasticity and dynamics of different scion–rootstock combinations in commercial mature tree production systems (e.g., Nonpareil versus Carmel scions grafted on the Nemaguard rootstock). The findings in this study provide tree crop researchers and the almond industry with a better understanding of *Prunus* root system resilience which can help to improve orchard production efficiency.

The findings on below-ground root system activity and tree water use under different reduced W and N input regimes corroborate the conclusions of Treeby [13], based on the same site, in that there is potential for inputs of both W and N to be reduced with minimal impact on yield. However, more experiments including reduced inputs and longer time frames with open collation of the results are needed to provide confidence in the adoption of lower-input, more resource-use-efficient orchard management practices. Additionally, further research into efficient and effective fertigation strategies is needed for growers to ensure that almond trees receive the right amount of water and fertilizer at the right time to enhance tree performance and fruit yield and quality. Furthermore, digital technologies for on-the-go assessment of tree stress and physiology may also assist. The best fertigation solution should integrate multivariate data sources, including data on the climate, soil, within-canopy microclimate, and tree performance, to support tree crop growers making real-time, in-time, and precautionary decisions.

## 4. Materials and Methods

### 4.1. Experimental Site, Plant Materials, and Treatments in the Commercial Orchard

The experiment was established at a commercial orchard (CMV Farms, Lindsay Point, Victoria, Australia) growing Nonpareil trees grafted on Nemaguard rootstocks. The experimental site was managed by Agriculture Victoria and described in detail by Treeby et al. [13]. To be brief, there were four treatments, including T1: farm standard irrigation and nitrogen (as urea) application (+W+N; 15 ML ha^−1^ water and 300 kg ha^−1^ nitrogen per season), T2: 46% reduction in nitrogen application (+W−N; 15 ML ha^−1^ water and 160 kg ha^−1^ nitrogen per season), T3: 30% reduction in irrigation (−W+N; 10.5 ML ha^−1^ water and 300 kg ha^−1^ nitrogen per season), and T4: 30% reduction in irrigation and 46% reduction in nitrogen application (−W−N; 10.5 ML ha^−1^ water and 160 kg ha^−1^ nitrogen per season). The treatments were applied factorially in a randomized complete block design with six replicate plots, leading to a total of 24 individual plots with a total area of 2.2 ha (Figure 1). The treatment levels for irrigation and fertigation were based on the prior literature reporting that a 30% reduction in water supply and a 46% reduction in nitrogen supply did not debilitate trees [21]. Muhammad et al. [21] reported that maximum kernel yield was reached at about 310 kg ha^−1^ nitrogen per season, above which the yield plateaued. High levels of nitrogen supply increase Nonpareil canopy density by stimulating vegetative growth [22]. These treatments could achieve an observable difference without causing a significant economic loss for the commercial farm while allowing the establishment of the trial and the modification of the site’s irrigation system [13]. Each plot consisted of eight trees and four rows, with two rows of Nonpareil trees alternating with Carmel and Monterey as pollinators. During the winter of 2015, irrigation and fertigation infrastructure was installed to enable the manipulation of both water (W) and nitrogen (N) supply in a structured manner.

### 4.2. Soil Sampling

The minirhizotron data were complimented with soil sampling (Figure 1) to allow the 3D root distribution to be determined. Soil sampling was only practical during the winter, due to the need to avoid damage to the root systems by the equipment. Samples were taken in each plot on five occasions, in July 2015 (prior to the onset of the treatments), August 2016, July 2017, August 2018, and August 2019. Typically, a three-core transect was taken, 1200, 2000, and 3500 mm from the tree line and approximately 400 mm away from a tree trunk along the tree line. In 2016, an additional fourth core was taken 700 mm from the tree line. Each core was taken at a 750 mm depth and split into three 250 mm sections.

The roots were extracted from each core section in the laboratory by washing and sieving, then split into two diameter categories: fine roots (<3 mm diameter) and coarse roots (>3 mm diameter). The fine roots were scanned for root length using WinRhizo (Regent Instruments, Ontario, Canada). Each root category was oven-dried to determine the dry mass.

### 4.3. Sap Flow Meter Installation and Observation

Sap flow sensors were installed to estimate the daily total water use of trees under the treatments. Data were typically collected from pre-budburst onwards, but the data reported are from shortly after budburst (1st September) until shortly before harvest (1st February) for consistency between trees and seasons. The tree water use of the same trees was determined each season using SFM1 sap flow sensors (ICT International, Armidale, NSW, Australia). These were trialed for part of the 2016/17 season and then used from budburst until harvest during the 2017/18 and 2018/19 seasons (Figure 1). Each of the 24 plots were installed with 1 sensor (i.e., 24 sensors in total). The sensor was installed midway between the soil surface and the emergence point (from the trunk) of the first tree branch (approximately 300 mm from ground level). The sensors were wrapped in foil insulation to minimize solar heating and prevent it affecting the measurements. The sensors had to be removed prior to harvest each season to prevent damage during tree shaking at harvest and could not be reinstalled until after a second shake. Bark depth, sapwood depth, and wood density were all determined on 5 mm diameter wood cores taken from trees adjacent to the experimental plots. Tree circumference was determined approximately once per season at the height at which the sensors were installed.

### 4.4. Statistical Analysis

All statistical analyses and principal component analyses were conducted in R (version 4.4.2), following Zhou et al. [11].

## 5. Conclusions

Root system resilience underpins stable almond yield under a 30% reduction in irrigation and/or a 46% reduction in nitrogen. The temporal dynamics of the treatment effects over 2017, 2018, and 2019 suggested a temporally strengthened +W−N effect to increase root biomass, average root diameter, specific root surface area (SRA), and specific root length (SRL) at deeper soil depths and at greater soil distance from driplines. These root acquisitive traits tended to be more sensitive to irrigation reduction than nitrogen reduction. The findings highlighted (1) the spatial (i.e., vertical and horizontal) and temporal responsiveness of root acquisitive traits and (2) their interconnections with tree water and nitrogen use efficiency, indicating a response coordination mechanism—at both the whole-root-system level and the whole-tree level—which could be valuable to be tested in future field-based studies.

## Data Availability

Data are available in Supplementary Table S1.

## Supplementary Materials

The following supporting information can be downloaded at https://www.mdpi.com/article/10.3390/plants15030409/s1, Table S1. Raw data of all traits on which PCA analysis was conducted, visualized in Figure 2, Figure 3 and Figure 4. Figures S1–S3. PCA depicted the differential responses of root traits under four treatments (T1: +W+N, T2: +W−N, T3: −W+N, T4: −W−N) along the vertical gradient (D1: 0–25 cm under the dripline; D2: 25–50 cm under the dripline; D3: 50–75 cm under the dripline; ‘0–75 cm’: the total of D1, D2, and D3) and along the horizonal gradient (dripline: 0 cm from the dripline; off-mound: 80 cm from the dripline; mid-row: 240 cm from the dripline) at the commercial farm at Lindsay Point, Victoria, Australia. Root traits included the specific root surface area (SRA), specific root length (SRL), average root diameter, root biomass, root length, and root surface area. Data were collected on roots (≤3 mm) from soil coring in the winters of 2017, 2018, and 2019.
