# Supplementary material for: Spatiotemporal Root-Trait Plasticity Underpins Almond Yield Stability and Enhanced Water and Nitrogen Use Efficiency Under Prolonged Fertigation Reduction
Source: Plants (Basel). 2026 Jan 29;15(3):409. doi: 10.3390/plants15030409 (PMC12899864; doi:10.3390/plants15030409)
Supplement: Supplementary file 1 [file plants-15-00409-s001.zip › Supplementary Figures_ Zhou et al. 2025.pdf]

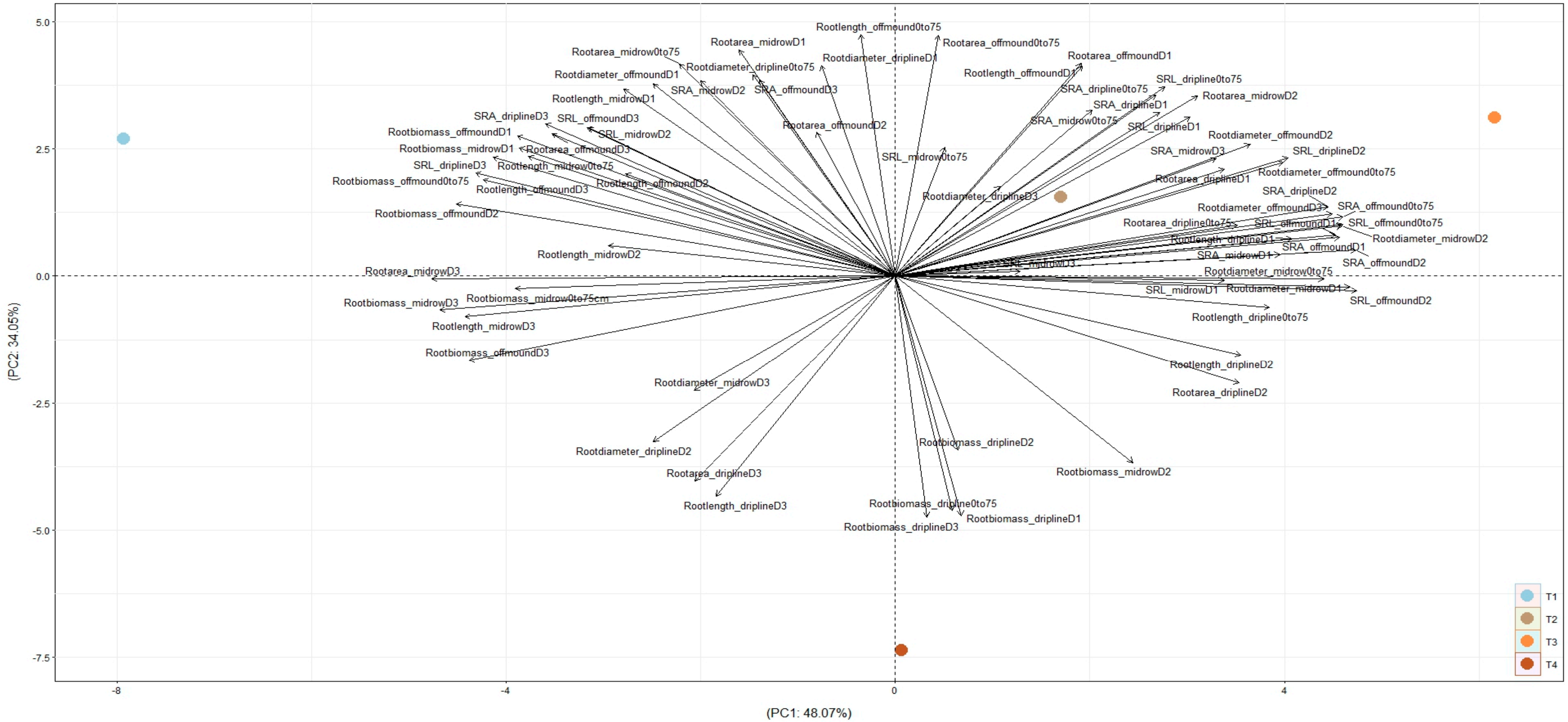

Figure S1. PCA depicted the differential responses of root traits under four treatments (T1: +W+N, T2: +W−N, T3: −W+N, T4: −W−N) along the vertical gradient (D1: 0–25 cm under the dripline; D2: 25–50 cm under the dripline; D3: 50–75 cm under the dripline; ‘0–75 cm’: the total of D1, D2 and D3) and along the horizonal gradient (dripline: 0 cm from the dripline; off-mound: 80 cm from the dripline; mid-row: 240 cm from the dripline) at the commercial farm at Lindsay Point, Victoria, Australia. Root traits included the specific root surface area (SRA), specific root length (SRL), average root diameter, root biomass, root length and root surface area. Data were collected on roots (≤ 3 mm) from soil coring in the winter of 2017 (after 2 years of treatments). PC1 explained 48.07% of the total variation and the PC2 explained 34.05% of the total variation. PC1 and PC2 explained 81.12% of the total variation.

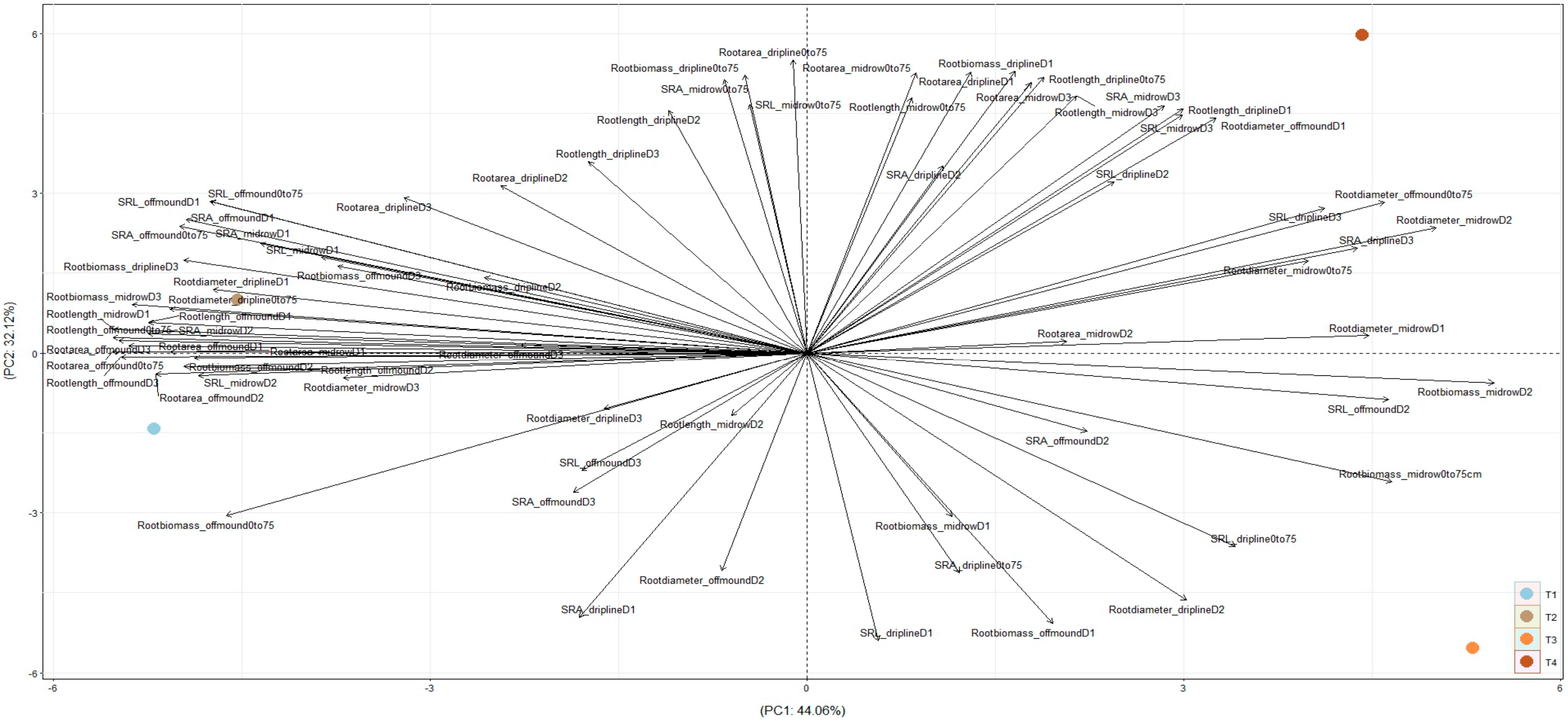

Figure S2. PCA depicted the differential responses of root traits under four treatments (T1: +W+N, T2: +W−N, T3: −W+N, T4: −W−N) along the vertical gradient (D1: 0–25 cm under the dripline; D2: 25–50 cm under the dripline; D3: 50–75 cm under the dripline; '0–75 cm': the total of D1, D2 and D3) and along the horizonal gradient (dripline: 0 cm from the dripline; off-mound: 80 cm from the dripline; mid-row: 240 cm from the dripline) at the commercial farm at Lindsay Point, Victoria, Australia. Root traits included the specific root surface area (SRA), specific root length (SRL), average root diameter, root biomass, root length and root surface area. Data were collected on roots (≤ 3 mm) from soil coring in the winter of 2018 (after 3 years of treatments). PC1 explained 44.06% of the total variation and the PC2 explained 32.12% of the total variation. PC1 and PC2 explained 76.18% of the total variation.

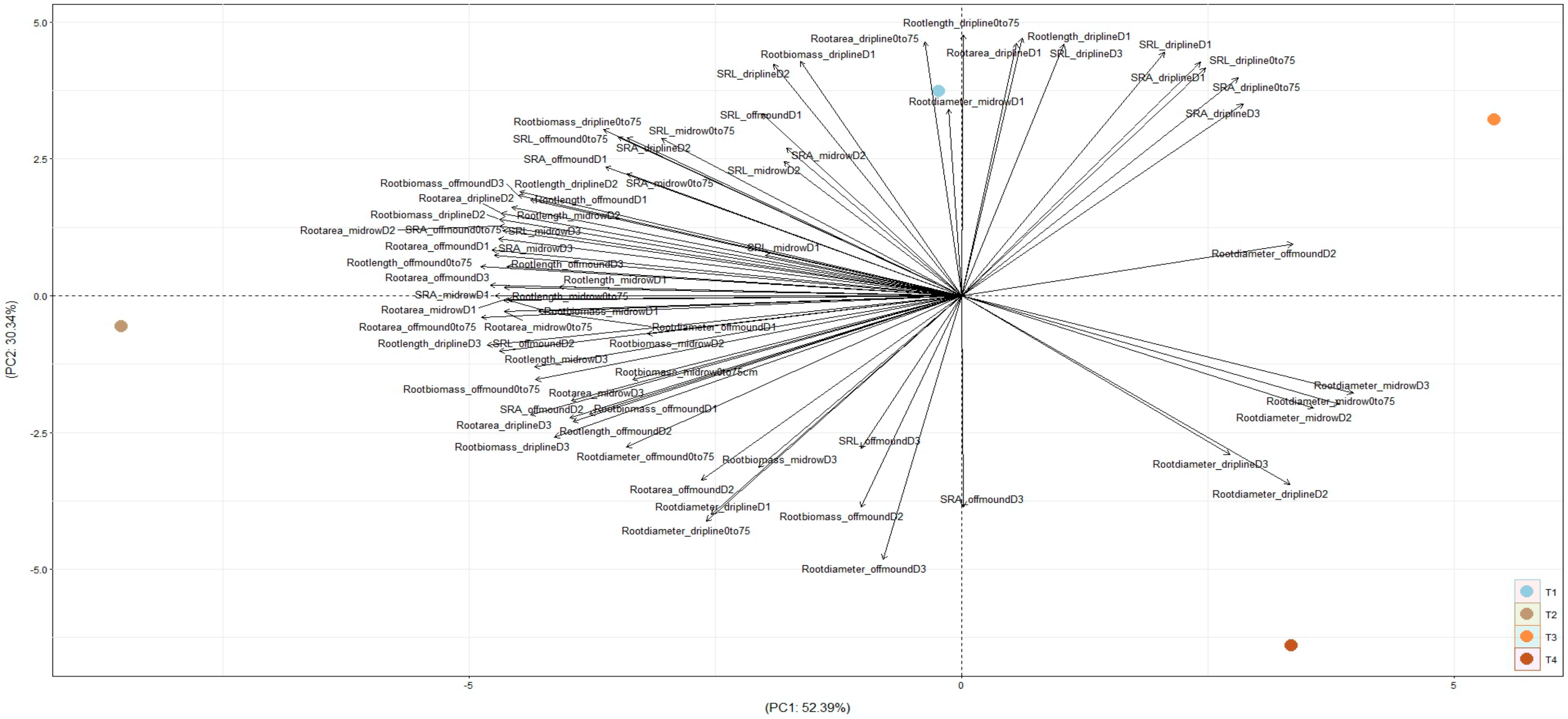

Figure S3. PCA depicted the differential responses of root traits under four treatments (T1: +W+N, T2: +W−N, T3: −W+N, T4: −W−N) along the vertical gradient (D1: 0–25 cm under the dripline; D2: 25–50 cm under the dripline; D3: 50–75 cm under the dripline; '0–75 cm': the total of D1, D2 and D3) and along the horizonal gradient (dripline: 0 cm from the dripline; off-mound: 80 cm from the dripline; mid-row: 240 cm from the dripline) at the commercial farm at Lindsay Point, Victoria, Australia. Root traits included the specific root surface area (SRA), specific root length (SRL), average root diameter, root biomass, root length and root surface area. Data were collected on roots (≤ 3 mm) from soil coring in the winter of 2019 (after 4 years of treatments). PC1 explained 52.39% of the total variation and the PC2 explained 30.34% of the total variation. PC1 and PC2 explained 82.73% of the total variation.
